# Relative HU profiling on clinical CT estimates the subchondral bone plate boundary: an ex vivo cadaveric mechanical validation study

**DOI:** 10.1038/s41598-026-56783-0

**Published:** 2026-06-08

**Authors:** Ryoya Shiode, Satoshi Miyamura, Satoshi Yamakawa, Toru Iwahashi, Hiroyuki Tanaka, Tatsuo Mae, Shoichi Shimada, Tsuyoshi Murase, Seiji Okada, Kunihiro Oka

**Affiliations:** 1 Department of Orthopaedic Surgery, Graduate School of Medicine, Osaka University, 2-2 Yamada-oka, Suita, 565-0871 Osaka Japan; 2 Department of Health and Sport Science, Graduate School of Medicine, Osaka University, Suita, Japan; 3 Department of Sports Medical Science, Graduate School of Medicine, Osaka University, Suita, Osaka Japan; 4 Osaka Keisatsu Hospital, Osaka, Osaka Japan; 5 Department of Neuroscience and Cell Biology, Graduate School of Medicine, Osaka University, Suita, Osaka Japan; 6 Department of Orthopaedic Surgery, Bellland General Hospital, Sakai, Osaka Japan

**Keywords:** Cadaveric study, Computed tomography, Hounsfield units, Mechanical indentation testing, Subchondral bone plate, Engineering, Health care, Medical research

## Abstract

The subchondral bone plate (SBP) is crucial for joint biomechanics and is implicated in degenerative joint disease. Absolute CT Hounsfield units (HUs) vary with scanner settings, limiting generalisability. We evaluated whether CT-derived relative HU profiling can estimate the location and thickness of the subchondral mineralized interface by comparison with mechanical indentation-derived measurements. Eighteen distal radii from nine formalin-fixed cadaver donors underwent low-dose CT and indentation testing. Post-test scans were registered to pre-test scans so CT- and indentation-based measures were sampled at identical locations. The SBP starting point and thickness were defined from HU attenuation transitions and load–displacement curves, respectively. Association between methods was evaluated with linear mixed-effects models using donor identity as a random intercept and summarised with marginal/conditional R²; agreement was descriptively assessed using Bland–Altman analysis. Inter-observer reliability was assessed using intraclass correlation coefficients. HU-based measures were strongly associated with indentation-derived measures, with fixed-effect slopes of 0.953 for the starting point and 0.988 for thickness, and R²m/R²c values of 0.916/0.918 and 0.912/0.912, respectively. Donor-level clustering effects were minimal. Reliability was good for HU-based measurements and good-to-excellent for indentation-based measurements. Bland–Altman analysis showed smaller bias and narrower limits of agreement for thickness than for the starting point. Relative HU profiling on standard clinical CT provided reproducible estimates of subchondral mineralized boundary features in this ex vivo cadaveric model. These findings support the feasibility of using relative HU transitions as a radiologic surrogate, while further histological and in vivo validation is required before clinical application.

## Introduction

The subchondral bone is an important structure that supports the articular cartilage and plays a vital role in load distribution and nutrient supply^1–4^. The bone is a dynamic organ that undergoes continuous remodelling throughout life, which involves osteoclastic bone resorption and mineralised bone removal, followed by new bone formation through osteoblasts to regulate bone metabolism^5^. This bone remodelling process allows bones to repair damage and adapt to mechanical loads^6,7^. Ageing^8–10^, joint surface morphology^11^, and conditions such as osteoarthritis are known to affect the structure and density of the subchondral bone.

Osteoarthritis is one of the most prevalent chronic joint diseases worldwide, affecting over 527 million people^12^. Earlier identification of joint degeneration remains challenging, and irreversible joint changes often follow subtle alterations in the subchondral bone^13^..

The subchondral bone microenvironment plays a pivotal role in the initiation and progression of osteoarthritis and osteoporosis. In osteoarthritis, the subchondral bone often becomes thickened and sclerotic beneath areas of cartilage degeneration, leading to increased bone volume and stiffness. By contrast, in osteoporosis, the subchondral bone plate tends to become thinner and less mineralised, compromising mechanical strength and increasing the fracture risk^14–22^. Such changes in the subchondral bone microenvironment may manifest as alterations in computed tomography (CT)-based Hounsfield unit (HU) profiles, reflecting relative differences in mineralisation, rather than providing a direct assessment of microstructural or biomechanical properties^14,23–28^..

High-resolution techniques such as high-resolution peripheral quantitative CT (HR-pQCT)^29–31^ and high-resolution magnetic resonance imaging (MRI)^32–35^enable the visualisation of the bone microarchitecture. Nevertheless, their clinical utility remains limited owing to cost and technical constraints. In contrast, CT is widely available^36,37^,and HUs from CT can be used to estimate bone density and strength^36,38^. However, HU values are not only scanner- and condition-dependent but also vary according to imaging protocols and patient-specific factors such as bone hydration and marrow content, limiting their generalisability^39–41^..

To address these limitations, we propose an approach that focuses on relative changes in HU values within a scan rather than on absolute HU thresholds. The current ex vivo cadaveric study aimed to evaluate whether relative HU profiles from standard clinical CT correspond to mechanically derived measurements of the subchondral mineralized interface at matched locations. Specifically, we examined whether voxel-to-voxel HU transitions could estimate the starting point and thickness of the subchondral mineralized boundary as defined by mechanical indentation. We hypothesised that relative HU transitions would correspond to the structural transition between cancellous bone and the mineralized subchondral interface. This approach may provide a reproducible radiologic surrogate for anatomical assessment of subchondral mineralized boundary features and serve as a basis for future histological and in vivo validation studies.

## Methods

### Study design and ethical approval

This cadaveric study was conducted in accordance with the principles embodied in the Declaration of Helsinki and was approved by the Institutional Review Board. All specimens were obtained with informed consent from donors or their legal representatives.

### Specimen preparation

Twenty wrists from 10 formalin-fixed cadavers were analysed. No visible damage was identified before testing, and none of the samples had post-fracture deformities or gross morphological abnormalities. Based on prior studies^39,42–44^, bilateral distal radii may exhibit asymmetry; however, because both radii were obtained from the same donors, donor identity was accounted for in the statistical analysis to address within-donor clustering. Two radii with osteoarthritic changes, as identified on radiographs by an orthopaedic hand surgeon, were excluded. Thus, the final sample included 18 radii from two male and seven female donors (age range: 75–102 years; mean: 89.2 years).

### Experimental workflow

**Fig. 1:**
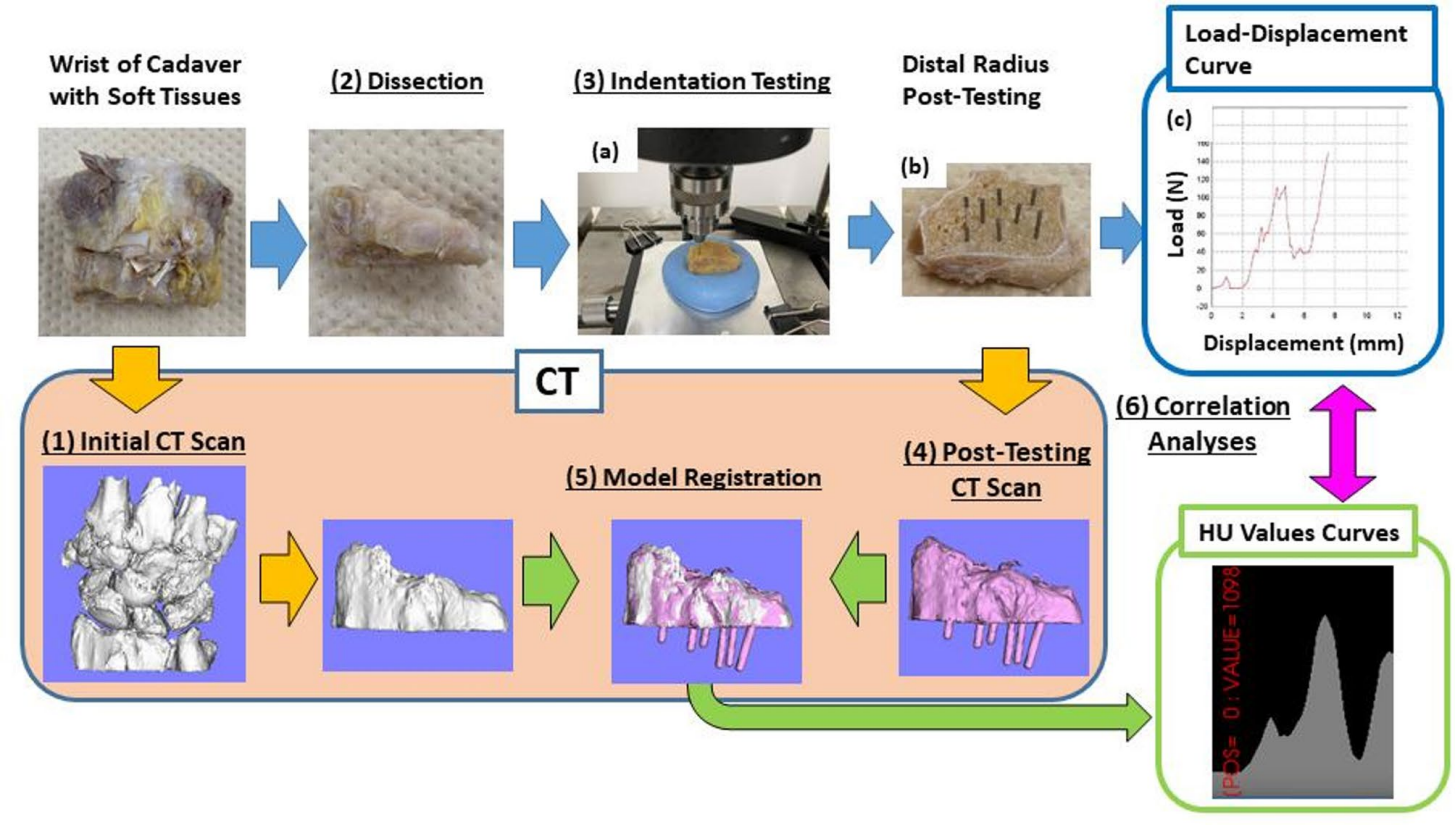
Overview of the experimental workflow. The six-step protocol was as follows: (1) initial computed tomography (CT) with the soft tissues left intact, (2) soft tissue removal, (3) mechanical indentation testing, (4) post-testing CT, (5) three-dimensional model registration, and (6) comparison between mechanical data and Hounsfield unit (HU) profiles from the initial scan. (**a**) Load-displacement curves were recorded using a universal testing machine with 1.0 mm rods. (**b**) Specimens were indented up to 150 N at 2 mm/min with 7–8 insertions. (**c**) One hundred eight valid measurements were collected from 140 trials.

Figure 1 shows an overview of the experimental procedure, which comprised the following steps: (1) Initial CT scans were acquired, with the skin being removed but leaving all subcutaneous soft tissues intact to simulate in vivo conditions and visualise the internal structure of the radius. (2) Mechanical dissection was conducted to remove all surrounding tissues while preserving bone integrity; the radius was then isolated for subsequent analysis. (3) Indentation testing was performed to quantify the mechanical strength of the subchondral bone. (4) Post-testing CT scans were obtained to identify the location where mechanical testing was performed. (5) For model registration, pre- and post-testing CT-derived bone models were superimposed to align the anatomical landmarks. (6) Finally, HU-based measurements derived from the initial CT scans were compared with mechanically derived measurements at the corresponding indentation sites.

### Indentation testing protocol

Load-displacement curves were obtained using a universal testing machine (Autograph AG-IS 500 N; Shimadzu, Kyoto, Japan). Stainless steel rods (diameter: 1.0 mm, length: 15 mm) were polished and mounted on a drill chuck and were subsequently secured within a sensor-equipped holder (force accuracy: ±1.5 N; displacement controlled at 2 mm/min) (Fig. 1a). The rods had flat, polished ends to standardise contact conditions.

Specimens were fixed to the testing platform using OSTRON II cement (GC Corporation, Tokyo, Japan) and cured for 30 min at 20 ± 1 °C and 50 ± 5% relative humidity. The articular surface was oriented downwards towards the cement to ensure consistent positioning.

The loading protocol (2 mm/min up to 150 N) was established based on preliminary trials demonstrating that a force of 150 N reliably resulted in the penetration of the subchondral bone plate on the articular surface of the distal radius. Rods remained in place after testing to preserve measurement geometry. Each specimen received 7–8 rod insertions at 12-mm intervals to avoid mechanical interference (Fig. 1b). Data were collected at 100 Hz using TRAPEZIUM software (Shimadzu) (Fig. 1c). Force and displacement were recorded from the testing machine (load cell and crosshead displacement). Out of 140 total measurements, 108 valid data points were retained after excluding 32 trials that failed to penetrate the articular surface. The distribution of excluded trials was reviewed descriptively to determine whether exclusions were concentrated in specific specimens or regions. The distribution of valid measurement sites was also summarized by anatomical region. Because the number of sites within each region was limited, regional analyses were considered descriptive and were not used for primary statistical inference.

### CT imaging and HU profile extraction

Helical CT scans were obtained using a low-dose protocol (Aquilion One; Canon, Tokyo, Japan): 120 kV, 30 mA, 0.625-mm slice thickness, and 0.48-mm in-plane resolution^45^. Consistent low-dose acquisition parameters were used in all scans to simulate clinical settings with minimised radiation exposure.

Initial CT scans were acquired before mechanical testing, with the soft tissues left intact. Post-testing CT scans were obtained after mechanical testing, with the soft tissues being removed.

**Fig. 2:**
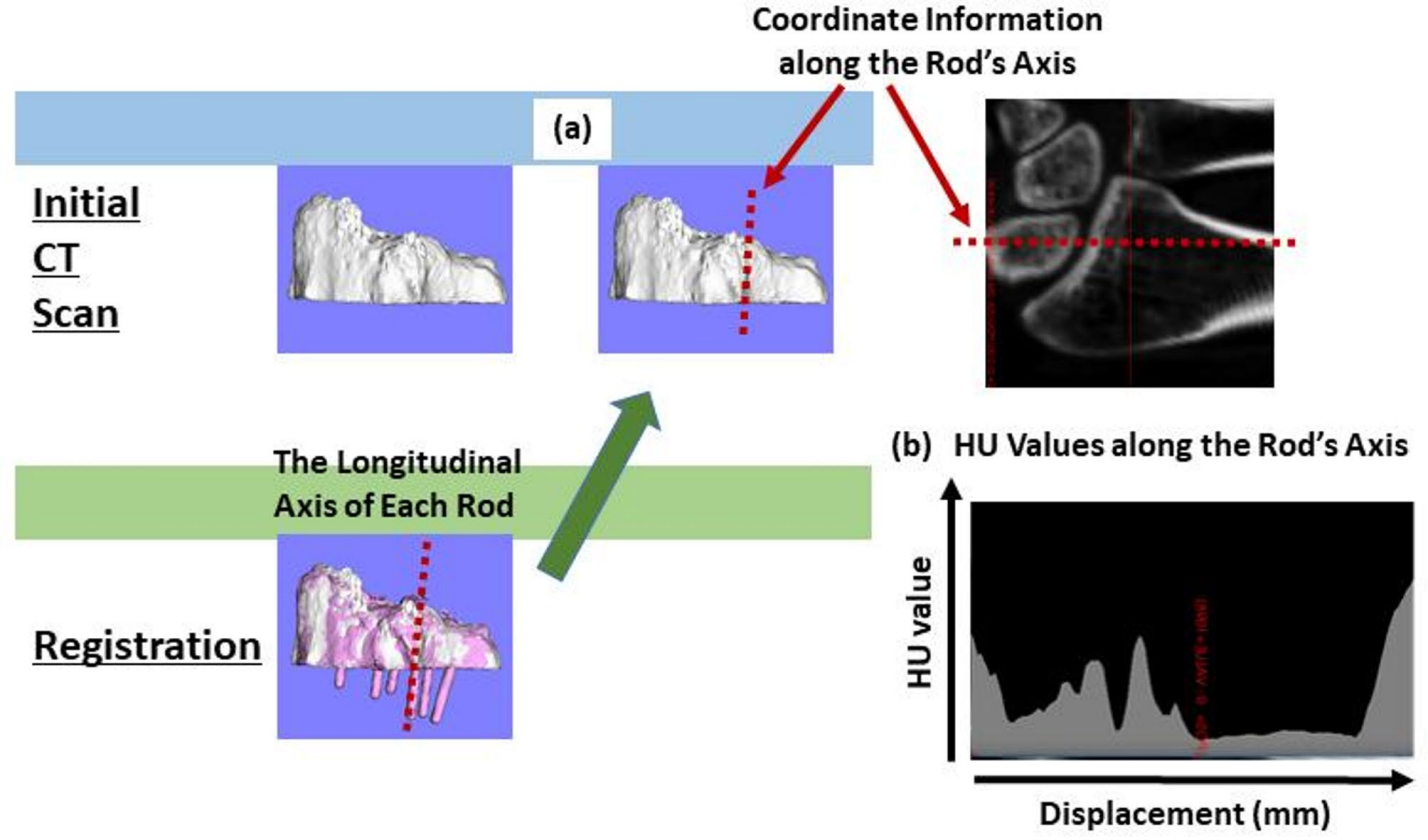
Computed tomography (CT)-based Hounsfield unit (HU) analysis and registration workflow. Bone models from pre- and post-testing CT scans were aligned using surface registration. Transformation matrices were applied to rod models to align the coordinate systems. HU values were extracted along the axis of each rod using BoneViewer software for density profiling.

CT data were exported in DICOM format. Three-dimensional reconstructions of the radii and rods were created with a 200-HU threshold using BoneViewer software (Teijin Nakashima Medical Co., Japan). Bone models from the two scans were registered using a surface-based iterative closest point algorithm^46^ implemented in Bone Simulator (Teijin Nakashima Medical Co.). The resultant transformation matrix was applied to the rod models to align them to the initial-scan coordinate system (Fig. 2a). Registration accuracy was visually confirmed using anatomical landmarks, and residual misalignment was expected to be within approximately one voxel. Subsequently, HU values were sampled along the longitudinal axis of each rod using BoneViewer software (Fig. 2b). HU sampling was performed at the native CT resolution (0.48 mm in-plane, 0.625 mm slice thickness), without sub-voxel interpolation. A single-voxel line profile was used to preserve spatial correspondence with the mechanical indentation trajectory, which represented a localized measurement along the longitudinal axis of each rod. Local area averaging could reduce image noise; however, it may also mix adjacent anatomical regions with different curvature and mineralization patterns, particularly in the distal radius articular surface. Therefore, we selected line-based sampling to match the local nature of indentation testing. Because HU sampling was conducted along a single-voxel line rather than an area-equivalent cylindrical volume, partial volume effects and image noise may have influenced the exact HU gradient, particularly near thin regions of the subchondral mineralized interface.

### Correlation between HU-based and mechanical measurements

**Fig. 3:**
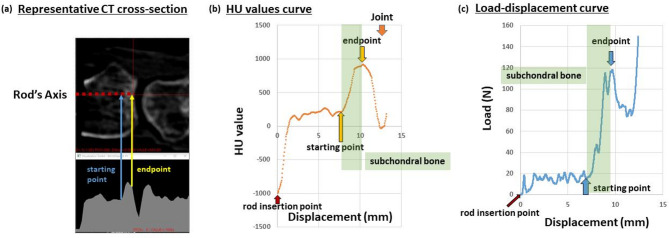
Definition of subchondral bone boundaries. Subchondral bone starting points and thickness were identified using (**a**) Representative CT cross-section showing the sampling path (dashed line) along the longitudinal axis of the indentation rod. The starting point and endpoint identified from the HU profile are indicated. (**b**) Hounsfield unit (HU) gradients and (**c**) mechanical resistance profiles. Two distances were calculated: (1) from the cortical reference to the onset and (2) from the onset to the endpoint (thickness), followed by comparison between HU-based and mechanically defined measurements.

The subchondral bone starting point and thickness were defined independently using two methods (Fig. 3):

- *HU-based definition*: The starting point was identified at the voxel where HU values began to increase from the cancellous bone, whereas the endpoint was identified at the voxel where HU values began to decrease (Fig. 3a).
- *Mechanical definition*: The starting point was defined as the initial rise in resistance on the load-displacement curve, whereas the endpoint was defined as a sharp decline, indicating transition into the cartilage (Fig. 3b).

Two distances were measured for each rod: (1) from the rod insertion point to the subchondral bone starting point and (2) subchondral bone thickness, defined as the distance from the starting point to the endpoint. These distances were calculated for HU-based and mechanical measurements and compared to assess association (mixed-effects models) and agreement (Bland–Altman).

### Observer reliability analysis

To evaluate measurement reproducibility, inter-observer reliability was assessed using a subset randomly selected from 24 measurement sites. Two independent observers (R.S. and S.M., both with over 10 years of experience in musculoskeletal orthopaedics) independently identified the subchondral bone plate border using HU-based attenuation profiles and load-displacement curves obtained from the indentation method.

Observers were blinded to each other’s measurements and the results of the alternative method. Inter-observer agreement was quantified using the intraclass correlation coefficient (ICC(2,1), two-way random-effects model, absolute agreement). As the primary objective was to evaluate inter-observer reproducibility, intra-observer reliability was not assessed.

### Statistical analysis

Statistical analysis was performed using JMP Pro 14 (SAS Institute Inc., Cary, NC, USA) and R (version 4.3.2). Linear mixed-effects models were fitted using lme4, with marginal/conditional R² and ICC derived using MuMIn and performance, respectively. The association between HU-based and mechanically derived measurements was evaluated using linear mixed-effects models with donor identity included as a random intercept to account for within-donor clustering (*n* = 9 donors). Pearson’s correlation coefficients were additionally computed as descriptive statistics. Bland–Altman plots were generated descriptively to assess agreement between HU-based and mechanically derived measurements for both the starting point and thickness. The mean difference (bias) and 95% limits of agreement (± 1.96 standard deviation) were calculated for each measurement. Because repeated measurements were clustered within radii and donors, Bland–Altman analysis was interpreted descriptively, whereas primary inference was based on linear mixed-effects models accounting for donor-level clustering. Model performance was summarised using marginal and conditional R² based on variance partitioning, and the specimen-level ICC was derived from the variance components.

## Results

### Distribution of valid and excluded measurements

Of the 140 indentation trials, 108 were retained as valid measurements and 32 were excluded because the rod failed to penetrate the articular surface. The excluded trials showed no apparent concentration in a specific donor, radius, or anatomical region. Among the 108 valid measurement sites, 49 were located in the scaphoid fossa and 59 were located in the lunate fossa. Measurement differences were reviewed descriptively by anatomical region. No marked regional concentration of measurement error was observed between the scaphoid and lunate fossae.

###  Correlation between HU-based and mechanical strength-based subchondral bone starting points

The distance from the rod insertion point to the subchondral bone starting point was quantified using HU-based and mechanical strength-based approaches. The mean distance error between HU-based and mechanical starting points was 0.14 ± 0.66 mm.

**Fig. 4:**
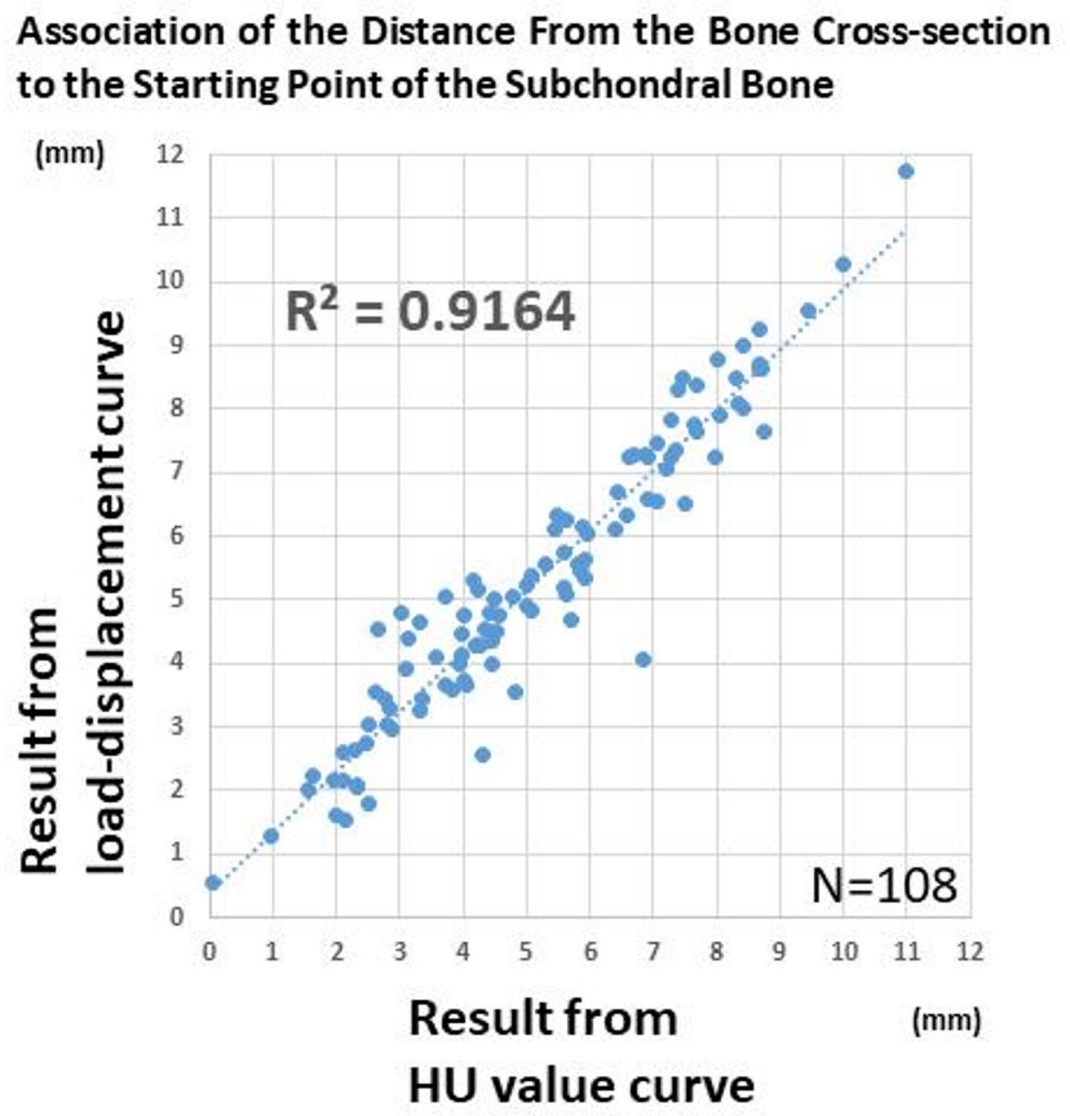
Association between Hounsfield unit (HU)-based and mechanically defined subchondral bone starting points. The distance from the rod insertion point to the onset of the subchondral bone plate was independently determined using relative HU transitions and indentation-derived load-displacement curves. Each point represents one measurement site (*n* = 108). The association was evaluated using a linear mixed-effects model with donor identity included as a random intercept. The fixed-effect slope was β = 0.953 (standard error = 0.030, 95% confidence interval: 0.894–1.012, *P* < 0.001), with marginal and conditional R² values of 0.916 and 0.918, respectively, indicating a strong association between HU-based and mechanically derived starting-point measurements.

In linear mixed-effects models accounting for donor-level clustering, HU-based measurements remained strongly associated with mechanically defined measurements. For the starting point, the fixed-effect slope was β = 0.953 (SE = 0.030, 95% CI: 0.894–1.012, *P* < 0.001), with marginal and conditional R² values of 0.916 and 0.918, respectively (Fig. 4). The donor-level ICC was 0.021, indicating minimal clustering effects. The limits of agreement for the starting point (− 1.16 to + 1.43 mm) were wider than those for thickness (− 0.47 to + 0.42 mm).

### Comparison of subchondral bone thickness

Subchondral bone thickness, defined as the distance from the starting point to the endpoint (the latter marked by a sharp decrease in HUs or resistance), was determined for both methods. The HU-based method yielded an average thickness of 2.08 ± 0.73 mm, whereas the mechanical strength-based approach produced a comparable value of 2.06 ± 0.76 mm. Sub-regional analysis demonstrated consistent agreement across both anatomical locations. In the scaphoid fossa (*n* = 49), the mean bias was + 0.030 mm with limits of agreement of − 0.475 to + 0.535 mm. In the lunate fossa (*n* = 59), the mean bias was + 0.015 mm with limits of agreement of − 0.379 to + 0.408 mm. No systematic sub-regional differences in measurement agreement were identified.

**Fig. 5:**
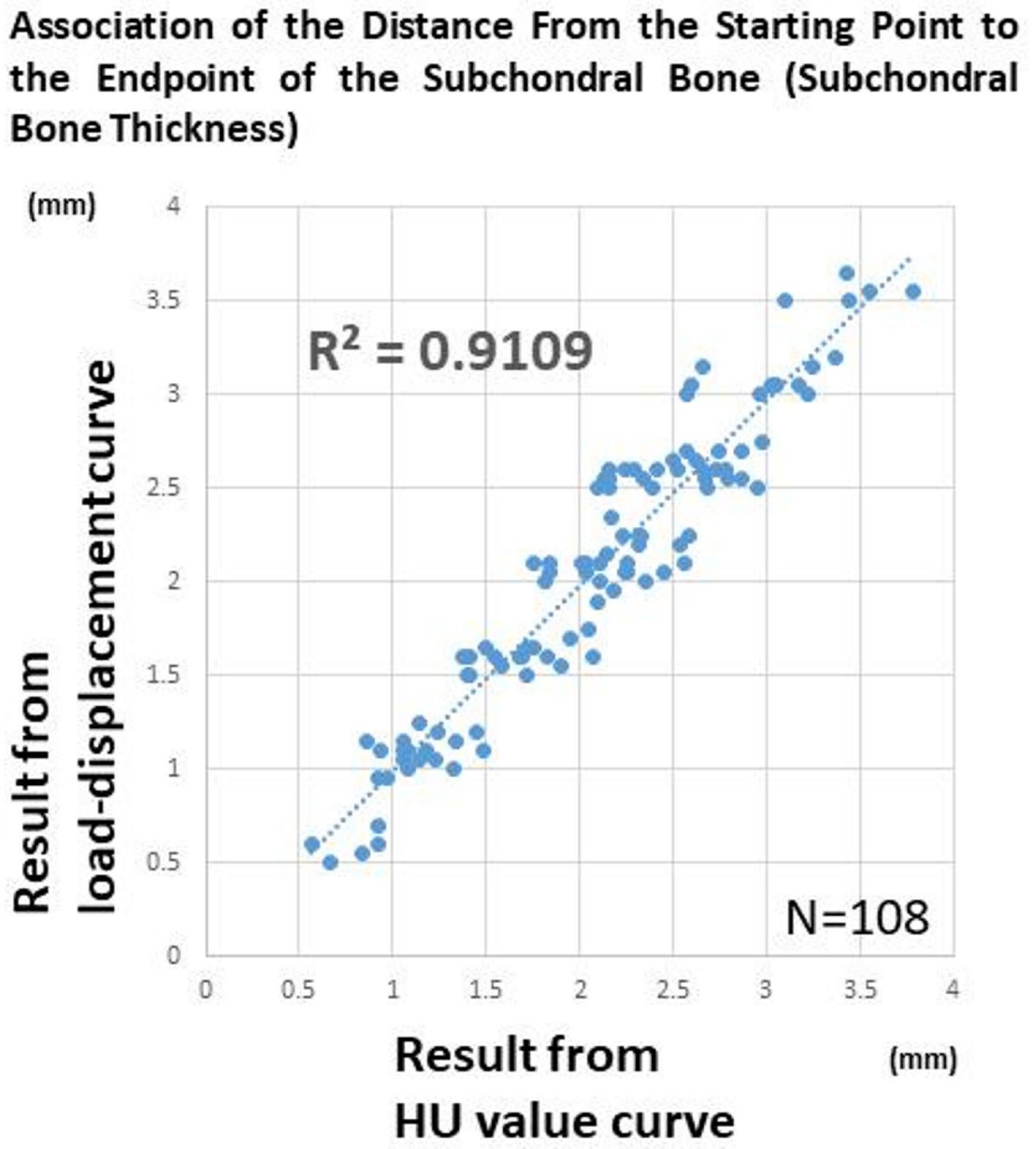
Association between Hounsfield unit (HU)-based and mechanically defined subchondral bone plate thickness. Subchondral bone thickness, defined as the distance from the starting point to the endpoint of the mineralised plate, was measured using HU-based attenuation profiles and indentation-derived mechanical responses. Each point represents one measurement site (*n* = 108). The association was analysed using a linear mixed-effects model with donor identity included as a random intercept. The fixed-effect slope was β = 0.988 (standard error = 0.032, 95% confidence interval: 0.925–1.051, *P* < 0.001), with marginal and conditional R² values of 0.912 and 0.912, respectively, demonstrating a strong association between HU-based and mechanical measurements.

In linear mixed-effects models accounting for donor-level clustering, HU-based measurements remained strongly associated with mechanically defined measurements. For the subchondral bone plate thickness, the fixed-effect slope was β = 0.988 (SE = 0.032, 95% CI: 0.925–1.051, *P* < 0.001), with marginal and conditional R² values of 0.912 and 0.912, respectively (Fig. 5). The donor-level ICC was 0.00, indicating minimal clustering effects.

### Inter-observer reliability

Inter-observer reliability analysis demonstrated good agreement for HU-based boundary identification (ICC(2,1) = 0.84, 95% CI: 0.68–0.93) and good-to-excellent agreement for indentation-based measurements (ICC(2,1) = 0.88, 95% CI: 0.63–0.95).

### Bland–altman analysis

**Fig. 6:**
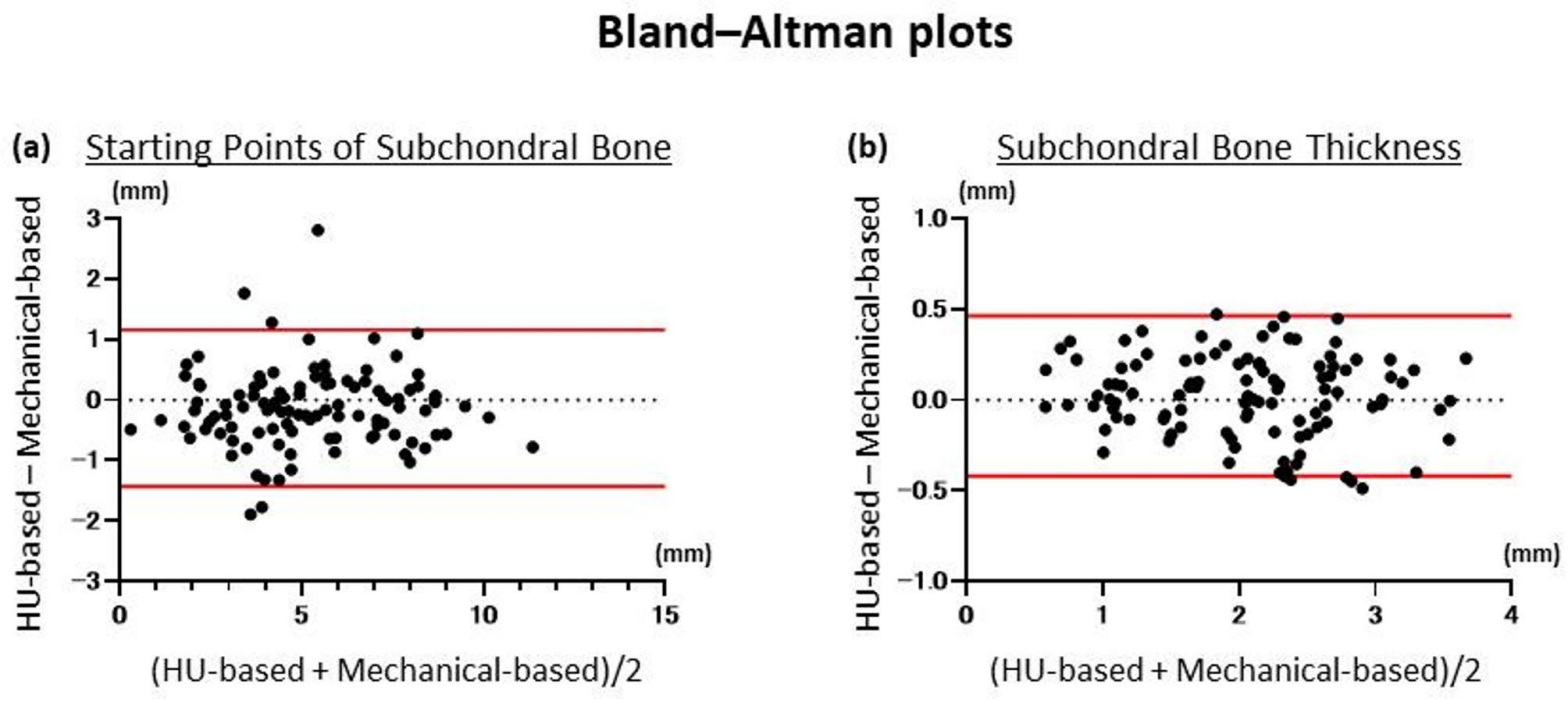
Bland–Altman analysis demonstrating agreement between Hounsfield unit (HU)-based and mechanically defined measurements. (**a**) Agreement for the subchondral bone starting point, defined as the distance from the rod insertion point to the onset of the subchondral bone plate. The mean difference (bias) was 0.13 mm, with 95% limits of agreement ranging from − 1.16 to + 1.43 mm. (**b**) Agreement for subchondral bone plate thickness, defined as the distance from the starting point to the endpoint of the mineralised plate. The mean bias was − 0.02 mm, with 95% limits of agreement of − 0.47 to + 0.42 mm. Solid lines indicate the mean difference, and dashed lines represent the 95% limits of agreement (± 1.96 standard deviation). The plots were interpreted descriptively because repeated measurements were clustered within radii and donors. Thickness showed smaller bias and narrower limits of agreement than the starting point.

Bland–Altman analysis was performed descriptively for both the starting point and thickness (Fig. 6). For the starting point, the mean bias was 0.13 mm, with 95% limits of agreement from − 1.16 to 1.43 mm. For thickness, the mean bias was − 0.02 mm, with narrower 95% limits of agreement from − 0.47 to 0.42 mm. Thus, the descriptive Bland–Altman results indicated smaller bias and less variability for thickness than for the starting point. Because repeated measurements were clustered within radii and donors, Bland–Altman plots were interpreted descriptively, whereas primary inference was based on mixed-effects models accounting for within-donor clustering.

## Discussion

### Summary of main findings

This ex vivo cadaveric study showed that relative HU measurements derived from standard clinical CT were strongly associated with mechanically derived measurements of the subchondral mineralized interface. The correspondence was more robust for thickness than for the starting point: thickness showed negligible bias and narrow limits of agreement, whereas the starting point showed wider limits of agreement despite strong association in the mixed-effects model. Therefore, HU-based thickness estimation may represent the more reproducible component of this approach, while the exact localization of the starting point should be interpreted more cautiously. Given the exploratory nature of this validation and the clustered structure of the data, these findings should not be interpreted as proving direct anatomical or histological equivalence; rather, they support the feasibility of HU-based radiologic estimation of subchondral mineralized boundary features.

### Comparison with prior literature

Previous studies have validated the relationship between absolute HU values and bone quality parameters, including mineral density and mechanical strength^38,40,41,47–49^. However, absolute HU values are strongly dependent on scanner type, reconstruction kernel, and imaging conditions, limiting their applicability across settings. Therefore, the present approach focuses instead on within-scan relative HU gradients to minimise such variability and provide a simple anatomical metric specific to each scan. Although HR-pQCT and high-resolution MRI provide excellent visualisation of microarchitecture^29–35^, they are costly and not widely available. In contrast, CT is ubiquitous^37^. Unlike HR-pQCT or micro-CT studies that directly evaluate trabecular structure or mineral density, our study does not attempt to infer microstructural or mechanical properties. Instead, it offers an incremental validation of a practical CT-derived marker that reflects the location and thickness of the subchondral bone plate.

This focus on boundary identification represents a more modest but clinically feasible contribution relative to prior microstructural investigations.

### Clinical relevance and reproducibility

The agreement between HU-based and mechanical measurements indicates that relative HU profiles serve as a repeatable objective method for evaluating the anatomical position and thickness of the subchondral bone plate. Because CT cannot differentiate between subchondral bone and calcified cartilage, and because partial volume effects affect thin structures, HU-based metrics should be interpreted strictly as estimates of the mineralised tissue boundary, rather than as measures of tissue quality. The spatial resolution of the CT protocol should also be considered when interpreting these measurements. The mean HU-based thickness in this study was approximately 2 mm, corresponding to roughly four in-plane pixels at an in-plane resolution of 0.48 mm and approximately three slice-thickness units at a slice thickness of 0.625 mm. This resolution is sufficient to capture a gross HU transition across the subchondral mineralized region, but it limits the precision with which sharp boundaries can be localized. In particular, partial volume averaging may blur the transition between cancellous bone, calcified cartilage, and the subchondral bone plate. Therefore, small differences in the estimated starting point or thickness should not be overinterpreted, and the present method should be regarded as a practical CT-based surrogate rather than a microscopic measurement of the true histological boundary.

In clinical practice, HU-based profiling may help to describe morphological characteristics of the subchondral plate on CT. Potential applications include preoperative planning for osteotomy procedures, where accurate characterisation of subchondral plate thickness may inform surgical decision-making, and longitudinal monitoring of subchondral changes in early osteoarthritis. However, its clinical applications require validation in vivo. In particular, ex vivo fixation and the absence of physiological loading may alter attenuation profiles and mechanical behaviour.

The HU-based boundary estimation demonstrated good inter-observer reliability, supporting the reproducibility of this approach despite the use of visually defined attenuation transitions. As expected, indentation-based measurements showed higher agreement, reflecting their mechanically defined nature.

The strong correspondence between HU-based and mechanically defined measurements persisted after accounting for repeated measurements within donors using mixed-effects modelling, indicating that the observed relationships were not driven by within-donor clustering. The consistency of agreement across the scaphoid and lunate fossae suggests that the method did not show obvious sub-regional bias within the evaluated regions of the distal radial articular surface. The distribution of valid measurement sites, with 49 sites in the scaphoid fossa and 59 sites in the lunate fossa, was anatomically reasonable because it broadly corresponded to the relative articular surface areas of these fossae reported in previous three-dimensional anatomical studies of the distal radius^50^. However, because the number of sites within each region was limited, regional differences were interpreted descriptively and no formal regional comparison was performed.

It is important to distinguish between the two outcomes examined in this study. Subchondral bone plate thickness showed more robust agreement, with a negligible mean bias of − 0.02 mm and narrow limits of agreement (− 0.47 to + 0.42 mm, total span 0.89 mm). In contrast, identification of the starting point yielded wider limits of agreement (− 1.16 to + 1.43 mm, total span 2.59 mm), suggesting that HU-based localisation of the plate onset carries greater uncertainty at the individual measurement level. This difference likely reflects the more gradual HU transition at the cartilage–bone interface, in contrast to the relatively abrupt decline marking the plate endpoint. Accordingly, thickness should be regarded as the more robust and reproducible output of this method, whereas starting-point localisation may require further refinement before clinical application.

### Limitations

First, indentation testing was performed on formalin-fixed radii. Formalin fixation has been shown to alter the mechanical properties of bone in a directional rather than random manner: fixed specimens demonstrate higher compressive failure loads than fresh controls, suggesting that fixation-induced cross-linking increases tissue stiffness and may shift the mechanically defined indentation onset to a higher load threshold^51,52^. This directional effect implies that mechanically defined subchondral plate boundaries in the present study may not be directly equivalent to those that would be identified in living bone. In contrast, CT-derived Hounsfield unit values reflect X-ray attenuation by mineralised tissue and are substantially less affected by formalin fixation: prior studies have reported that QCT-based bone mineral density and trabecular bone microstructure parameters remain stable following formalin fixation of up to six months^53^, and that bone mineral content measured by DXA shows only negligible changes after fixation (mean loss < 1%)^54^. Importantly, because both HU-based and mechanical measurements were obtained from the same fixed specimens in the present study, fixation-induced changes would affect mechanical measurements preferentially, potentially introducing a systematic offset from in vivo conditions without substantially degrading the relative agreement between the two methods. These findings should not be directly extrapolated to in vivo settings, and future studies using living subjects are required to confirm the clinical applicability of the proposed method. Second, indentation was conducted in air rather than physiological fluid, which may affect local mechanical resistance. Third, the donor cohort had a mean age of 89.2 years and was predominantly female, reflecting a population in which osteoporosis is prevalent. Osteoporotic bone is characterised by reduced bone mineral density and thinning of the subchondral bone plate, which may differ from the bone quality observed in the typical osteoarthritis patient, who tends to be younger and may exhibit subchondral bone sclerosis rather than atrophy. This age and sex bias may therefore limit the generalisability of these findings to younger or mixed-sex OA populations. However, it should be noted that the relative HU profiling approach employed in this study does not rely on absolute attenuation thresholds, and the definition of subchondral plate boundaries is based on local HU transitions rather than absolute bone density values. This methodological feature may partially mitigate the impact of osteoporotic bone quality on measurement performance, though this remains to be confirmed in cohorts more representative of clinical OA populations. Fourth, the 108 measurement sites were nested within 18 radii from only 9 donors, meaning that the true biological sample size is small (*n* = 9 donors). Readers should be cautioned against interpreting the site count as equivalent to a large number of independent observations, as measurements within the same donor share underlying biological characteristics. Data points were therefore not statistically independent, and agreement estimates should be interpreted in the context of this clustered design. Primary inference was based on linear mixed-effects modelling with donor identity as a random intercept to account for within-donor clustering; however, the small number of donors limits the precision of variance component estimates and may affect the generalisability of the findings. Finally, as CT-based HU cannot distinguish calcified cartilage from subchondral bone, the boundary determined through HU transitions likely encompassed both structures, thus representing an anatomical-radiologic interface, rather than a histological definition.

## Conclusion

Relative Hounsfield unit profiling demonstrated strong association with mechanically defined subchondral mineralized interface thickness in this cadaveric study, with negligible bias and narrow limits of agreement. Identification of the subchondral bone plate starting point showed acceptable but weaker agreement, and should be interpreted with greater caution pending further methodological refinement. The method provides a reproducible means of assessing subchondral mineralized interface thickness on standard clinical CT and represents an ex vivo proof-of-concept for boundary localisation. These findings should be interpreted in the context of the small biological sample size (9 donors, 18 radii), and replication in larger prospective in vivo studies is required before clinical translation. Although clinical utility remains to be established, the technique may serve as a structural reference for investigating joint degeneration and may support surgical research requiring anatomical characterisation of the subchondral bone.

## Data Availability

Data supporting the findings of this study are available from the corresponding author upon reasonable request.
